# Stimuli-responsive injectable chitosan-based hydrogels for controlled drug delivery systems

**DOI:** 10.3389/fbioe.2022.1126774

**Published:** 2023-01-06

**Authors:** Hamidreza Garshasbi, Saba Salehi, Seyed Morteza Naghib, Sadegh Ghorbanzadeh, Wei Zhang

**Affiliations:** ^1^ Nanotechnology Department, School of Advanced Technologies, Iran University of Science and Technology and Biomaterials and Tissue Engineering Department, Breast Cancer Research Center, Motamed Cancer Institute, Iran University of Science and Technology (IUST), The Academic Center for Education, Culture and Research (ACECR), Tehran, Iran; ^2^ State Key Laboratory of Structure Analysis for Industrial Equipment, Department of Engineering Mechanics, Dalian University of Technology, Dalian, China

**Keywords:** injectable, hydrogels, chitosan, stimuli-responsive, pH

## Abstract

In the last decade, injectable hydrogels have attracted a lot of attention due to their excellent functional properties in the field of drug delivery for precise, non-invasive and focused tissue locations. Therefore, designing drug delivery systems (DDS) responsive to hydrogel stimuli to release a drug to an external stimulus with various advantages, can be very challenging. Due to their biocompatibility, mucosal adhesion, and hemostatic activity, chitosan (Chitosan)-based hydrogels offer a lot of potential for tissue engineering and drug delivery. It can be difficult to manage the structure of these stimuli-responsive CS hydrogels or they may require additional crosslinking agents, such as hydrogels with dual pH and thermo-responsiveness. Therefore, it is crucial to create these hydrogels for medicinal applications.

## 1 Introduction

Potential delivery methods for controlled release of bioactive substances include injectable polymer hydrogels. Numerous stimuli, including pH, temperature, light, enzymes, and magnetic fields, can cause these polymers’ sol-to-gel transitions in aqueous solutions to change. At room temperature, the low-viscous polymer solution may be conveniently combined with therapeutic agents, such as chemotherapeutics, protein medicines, or cells. Through the use of a syringe or catheter, therapeutic agent-containing solutions may be easily injected into the desired locations (Thambi et al., 2016).

Researchers have focused on developing the optimum drug delivery system (DDS) that can adapt to various physiological needs, such as liposomes, microspheres, nanoparticles, polymeric micelles, and hydrogels. The most prevalent form of responsive nano-system for cancer medication delivery is one that responds to pH variations, owing to the fact that tumors have extracellular pH values that are lower than those of healthy tissues and the circulation (pH = 7.4) (Andrade et al., 2021). More crucially, injectable hydrogels with mucoadhesive qualities may be tailored to a particular region because they attach to a specific spot in the mucosa. This prevents medication diffusion, which may significantly increase treatment efficacy and lessen adverse effects (Liang et al., 2019).

Chitosan (CS) is a polysaccharide identified as suitable for drug/gene delivery depots (Andrade et al., 2021). Due to its advantageous characteristics, including non-toxicity and biodegradability, it has been widely formed into various hydrogels for the administration of ophthalmic drugs. It is the second most prevalent natural polysaccharide after cellulose. (Lin et al., 2019). CS is generally prepared by partial deacetylation of chitin, which is found in the exoskeletons of arthropods, such as crabs, shrimps, and lobsters. It has been researched in mucoadhesive delivery because its cationic character shows mucoadhesive qualities. CS hydrogels are formed by connecting hydrophilic polymer chains. Injectable CS hydrogels have lately been used to transport medications as well as produce bone, cartilage, and nerve tissue (Thambi et al., 2016).

## 2 Cartilage healing

An injectable carboxymethyl chitosan-oxidized chondroitin sulfate hydrogel (CMC-OCS) hydrogel containing microspheres (MPs) was made using the Schiff base crosslinking procedure. The kartogenin (KGN) loaded poly (lactide-co-glycolic acid) MPs (MPs/KGN) were made using the PME method. *In vitro*, the obtained CMC-OCS/MPs/KGN showed slower weight loss and a shorter gelation time than control hydrogels. The hydrogel/MPs’ compressive elastic modulus was greatly enhanced, which is good for cartilage healing. While waiting, the PLGA MPs/KGN might release KGN in regulated bursts in response to ultrasound. *In vitro* rabbit bone marrow mesenchymal stem cells (rBMMSCs) sown in the scaffolds demonstrated noticeably enhanced vitality throughout a 7–10-day culture (Yuan et al., 2021).

**FIGURE 1:**
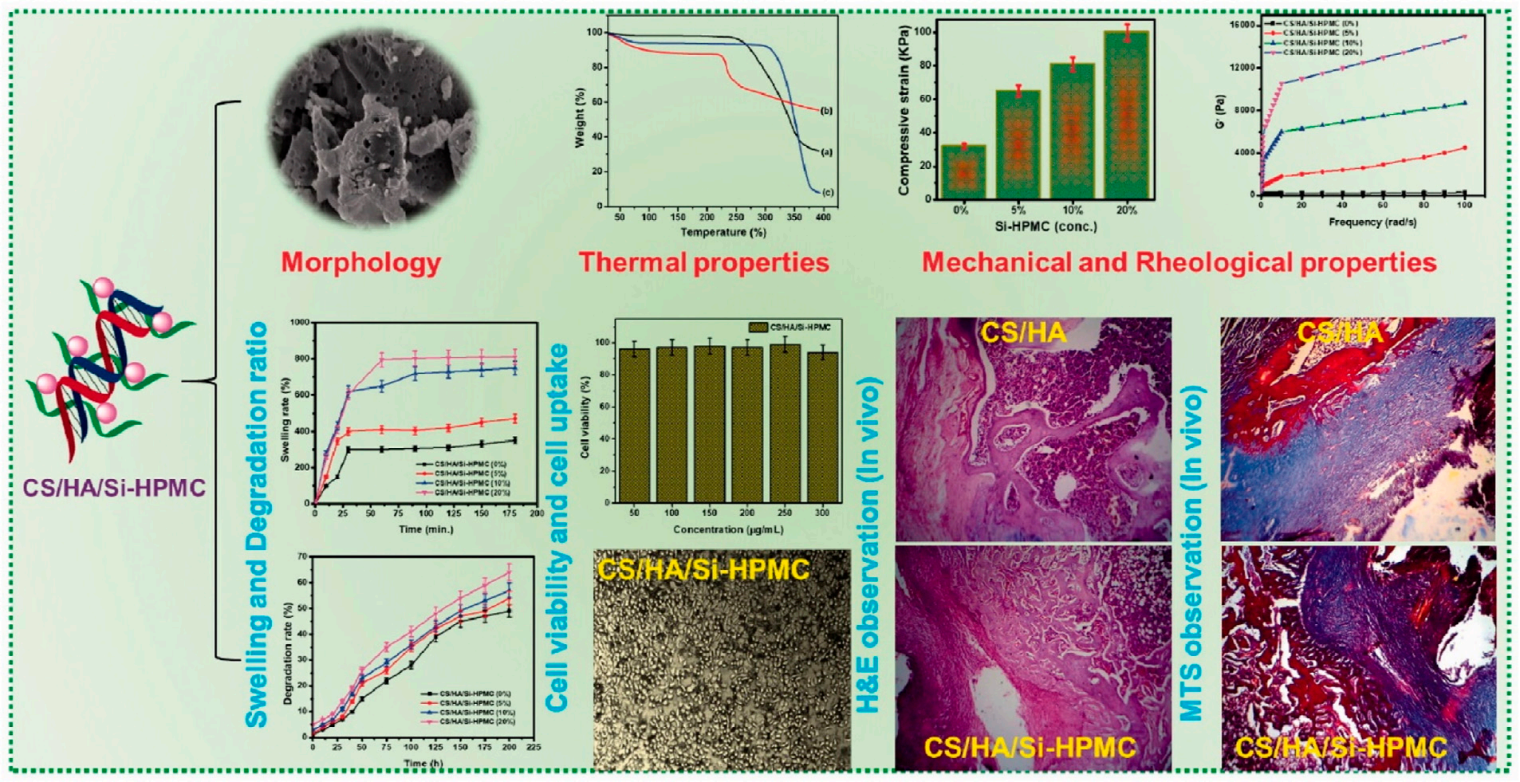
Schematic representation of the synthesis process of Si-HPMC incorporated CS/HA injectable hydrogel. The regeneration rate of the CS/HA/Si-HPMC (3%) was almost 79.5% at 21 days for long retention periods, demonstrating relatively good *in vivo* bone regeneration (Hu et al., 2021) (Open access).

A novel injectable hydrogel composite made of water-soluble CS/HA and silanized hydroxypropyl methylcellulose (Si-HPMC) was created for cartilage tissue creation (Figure 1). Si-HPMC was uniformly distributed all across CS/HA hydrogel system and had good impact on the mechanical properties by stabilizing the hydrogel network, and lowering weight loss at the expense of quickening the gelation process and enhancing the swelling ratio. The addition of SiHPMC (3.0%) in the CS/HA hydrogel had an effect on the surface characteristics and pore size of the composite scaffolds. The physicochemical characteristics of the hydrogel network, including its shape, surface properties, swelling rate, degradation behavior, and mechanical, rheological, and compressive capabilities, may be controlled by varying the weight ratio and concentration of the CS/HA hydrogel and Si-HPMC. Additionally, L929 cells were active and successfully multiplied on the CS/HA/Si-HPMC hydrogel throughout the *in vitro* cell culture period. One of the vital components of cartilage, HA, may be the result of this biocompatibility. In fact, when seeded in the composite CS/HA/SiHPMC hydrogels, the *in vitro* chondrocyte survival up to 21 days of culture is much higher. These results demonstrate that the CS/HA/Si-HPMC hydrogels, especially the one containing 3% (w/v) Si-HPMC, exhibited the best overall properties for use in bone regeneration. When coupled with chondrocytes in subcutaneous or nude mice, CS/HA/Si-HPMC hydrogels have been shown to be useful for cartilage tissue engineering. As a result, the injectable CS/HA hydrogel loaded with Si-HPMC is a promising tissue engineering approach with a lot of clinical cartilage tissue healing potential (Hu et al., 2021).

## 3 Injectable hydrogels for dental pulp stem cells

According to Samiei et al., the hydrogels that incorporate more gelatin have a somewhat looser network than the others. The hydrogel with less gelatin has a slightly higher value of G′, which denotes more elasticity as a result of more CS amine groups being crosslinked through a covalent connection with genipin. Since there were very few dead cells in any of the hydrogels, this demonstrated the remarkable biocompatibility of the made hydrogels for human dental pulp stem cells (hDPSCs). After 21 days, hDPSCs cultivated on produced hydrogels showed a considerable increase in calcium deposition, as shown by the quantitative findings of alizarin red staining. Furthermore, the greatest Alkaline phosphatase (ALP) activity was shown by hDPSCs cultivated on hydrogel that included more gelatin. After 21 days, the hydrogel with more gelatin expressed late osteogenic genes such OCN and BMP-2 6 and 4 times, respectively, more than the control group. The Poly (N-isopropylacrylamide) (PNIPAAm-g-CS) copolymer/gelatin hybrid hydrogel that was created had excellent properties and produced the osteogenic differentiation required for dental tissue engineering (Samiei et al., 2022).

## 4 Drug delivery system

Focusing on the positive benefits of Fmoc-FF self-assembly and its electrostatic interaction with glycol chitosan (GCS), a straightforward method for producing injectable self-healing hydrogels for Doxorubicin (DOX) administration has been reported. The final product, a hydrogel-based technology, showed a novel Ph-responsive DOX release, with the drug release mechanism being improved in moderately acidic environments. This finding may pave the way for controlled drug release in a typical, moderately acidic tumor microenvironment. The new hydrogel was undoubtedly a successful method for the controlled release of DOX, given the slow rate of release of DOX under physiologically normal settings. Notably, the DOX-loaded hydrogel was efficiently cytotoxic to the human tumor cell line A549 in a test tube (Shim et al., 2021).

Physical mixing of CS with Pluronic-F127 (PF) produced the hydrogels. Tripolyphosphate (TPP) was further utilized as a crosslinking agent. In comparison to pluronic gel alone, the gel duration was lengthened by the addition of CS. The morphology of the hydrogels generated was altered by the addition of TPP to the substance. To test whether hydrogels were harmful to human chondrocytes, MTS and Live/Dead^®^ assays were conducted. Dexamethasone (DMT) was released *in vitro* from the CS-PF and CS-PF-TPP gels over a longer period of time than the PF hydrogel. Studies conducted *in vivo* revealed that hydrogels kept the fluorescent component in the joint longer than when it was given in Phosphate-buffered saline (PBS) alone. These findings imply that the DMT-loaded CS-PF and CS-PF-TPP hydrogels may provide an effective drug delivery system for the osteoarthritis treatment (García-Couce et al., 2022).

According to the contact angle measurement, the chemically altered chitosan exhibits a less hydrophilic character than pure chitosan, which results in poor swelling in an aqueous environment. The hydrogel is made from graft copolymer, and because of the hydrogel’s porous network-like structure, the drug molecules are shielded from their hostile environment. In comparison to pure chitosan, the graft copolymer has the ability to deliver the medicine in a sustained way, according to an *in vitro* drug release study. Graft copolymer is discovered to have a significantly higher rate of cellular absorption than pure medication, making it a more effective delivery method. The possibility of using the graft copolymers as an injectable hydrogel has been suggested by an *in vivo* gelation investigation in a rat model (Mahanta and Maiti, 2019).

## 5 Bone tissue engineering

To overcome the limitations of CS/β-Glycerophosphate (GP) hydrogel, a thermosensitive, injectable halloysite nanotubes (mHNTs)/CS/GP NC hydrogel was developed. The objective was to develop an injectable nanocomposite (NC) CS hydrogel containing modified mHNTs. This research enhances the mechanical robustness of the resultant scaffold mHNTs as well as the proliferation of human adipose tissue-derived stem cells (hASCs) within it. Overall, the results for bone differentiation showed that IC/mHNTs improved the mechanical strength of CS hydrogel and enhanced the difference of encapsulated hASCs into bone tissue because of their stiffness, tubular structure, and ability to dynamically deliver IC as an osteogenic inducer agent (Kazemi-Aghdam et al., 2021).

For tissue engineering applications, the optimal chitosan/oxidized-modified quince seed gum/curcumin-loaded (CS/OX-QSG) hydrogel (ratio of 25:75) with varied curcumin-loaded in halloysite nanotubes (CUR-HNT) levels was effectively synthesized. The hydrogel with the highest OX-QSG concentration (CS/OX-QSG 25:75) exhibited a spongy structure with bigger and smaller holes as well as a high crosslinking density. Because of their good mechanical and anti-bacterial capabilities, the results demonstrated that CS/OX-QSG hydrogels with a 25:75 ratio and 30% CUR-HNTs may provide a viable scaffold for tissue engineering applications (Yavari Maroufi and Ghorbani, 2021).

Using the enzymatic crosslinking process of horseradish peroxidase, Jung et al. created *in situ* forming CS/poly (ethylene glycol) (PEG) hydrogels with better mechanical characteristics. By altering the quantity (0%–100%), molecular weight (4, 10 and 20 kDa), and of the PEG derivatives, the impact of PEG on the physico-chemical characteristics of hybrid hydrogels was extensively clarified. The resultant hydrogels, which are equivalent to commercially available fibrin glue, showed outstanding hemostatic activity and are extremely biocompatible *in vivo* (Jung et al., 2021).

Kaur et al. have created injectable hydrogels that are mechanically strong and have enhanced osteogenic properties. The sol-gel transition occurs at 37 C, which is the physiological temperature, and all of the hydrogels have been found to be thermos-responsive. Due to the hydrogel components’ positively and negatively charged groups, which attract Ca2+ and PO43 to form hydroxyapatite (Hap) on the surface, all of the hydrogels were demonstrated to be bioactive after only 1 day of incubation in simulated body fluid (SBF). Incorporating carboxylated single wall carbon nanotubes (COOH-SWCNTs) with CS and collagen (Coll) by the application of -GP increased cross-linking, created the greatest possible thermos-responsive and injectability characteristics, and significantly enhanced mechanical properties. Additionally, the hydrogels with COOH-SWCNTs incorporated significantly increase cell proliferation and support osteogenic differentiation as compared to pure CS/Coll (Kaur et al., 2021).

Taymouri et al. successfully developed a chitosan/Silk fibroin (CS2/SF0.5) thermosensitive hydrogel containing dipyridamole loaded polycaprolactone nanoparticles (DIP-PCLNPs) as a novel and non-invasive local drug delivery approach for bone tissue engineering. DIPPCL NPs were produced using the solvent-emulsification evaporation method, and the formulation factors were optimized using an irregular factorial design. The optimal conditions for producing DIP-PCL NPs were 7 mg DIP, 1.5% PVA, a W/O volume ratio of 4, and a 4-min sonication period. This perfect formulation was put into the CS2/SF0.5 thermosensitive hydrogel. Since the results demonstrated that the amount of the inserted SF altered the network structure of the CS hydrogel, the gel scaffolds containing 0.5% SF had a shorter gelation time and stronger compressive strength when compared to other composite hydrogels. In an *in vitro* cell culture examination, the DIP-PCL NPs- CS2/SF0.5 hydrogel shown the greatest capacity to encourage the proliferation and activity of MG-63 cells, as demonstrated by a significant increase in cell viability, ALP activity, and calcium deposition (Taymouri et al., 2021).

In order to increase cardiac tissue’s functioning, cardiac tissue engineering supports, replaces, or repairs it. Application of the polysaccharides—of which chitosan is a key component—addresses the main problem with the vitality of the implanted cells. Chitosan aids in the provision of mechanical support, prevents the spread of pro-inflammatory chemicals, and encloses bioactive elements beneficial for the regeneration of heart tissue. Chitosan’s positive charge and hydrophilicity, among other properties, enable the development of a soft tissue milieu, particularly when combined with biomolecules. Scaffolds made of chitosan support stem cell proliferation and differentiation by providing mechanical strength (Patel et al., 2021).

## 6 Anti-cancer

Injectable polysaccharide hydrogels that are biocompatible and self-healing have been produced by the first-ever chemical crosslinking of multialdehyde guar gum (MAGG) with numerous aldehyde groups and N, O-carboxymethyl chitosan (N, O-CMCS) *via* pH-sensitive, biodegradable, and dynamic Schiff base connections. These hydrogels were ideal for injectable drug administration because of their exceptional viscoelastic, thixotropic, and self-healing properties. After Dox loading for 5 days, these hydrogels demonstrated a pH-responsive release, with a higher release at tumoral pH than at physiological pH. MTT and hemolytic assays demonstrated the non-toxicity of these hydrogels. The Dox-loaded hydrogel significantly reduced MCF-7 cell viability, with 72% of the cells dying (Pandit et al., 2021).

To generate DOX@CSSH/HNTs-SH Gel, thiol group-modified halloysite nanotubes (HNTs) were first loaded with DOX before being added to the gel precursor. This gel loaded and gradually released water-soluble DOX to lessen the cytotoxicity of thiolated chitosan (CSSH) Gel while also enhancing its mechanical characteristics. The results showed that the HNTs-SH could be evenly distributed throughout the gel matrix to boost the gel’s compressive strength and that the gel was pH sensitive to release DOX quickly in the tumor’s acidic microenvironment, where MCF-7 cells could take it up and effectively inhibit MCF-7 cell growth. According to *in vivo* tumor resection and recurrence inhibition experiments, the gel may stop lung metastasis, lessen tumor recurrence, improve survival rates, and mend surgically injured tissues. The *in-situ* injectable DOX@CSSH/HNTs-SH Gel may be developed as a novel drug delivery system to inhibit tumor recurrence and heal surgical wound tissues after tumor removal (Li et al., 2021).

**FIGURE 2:**
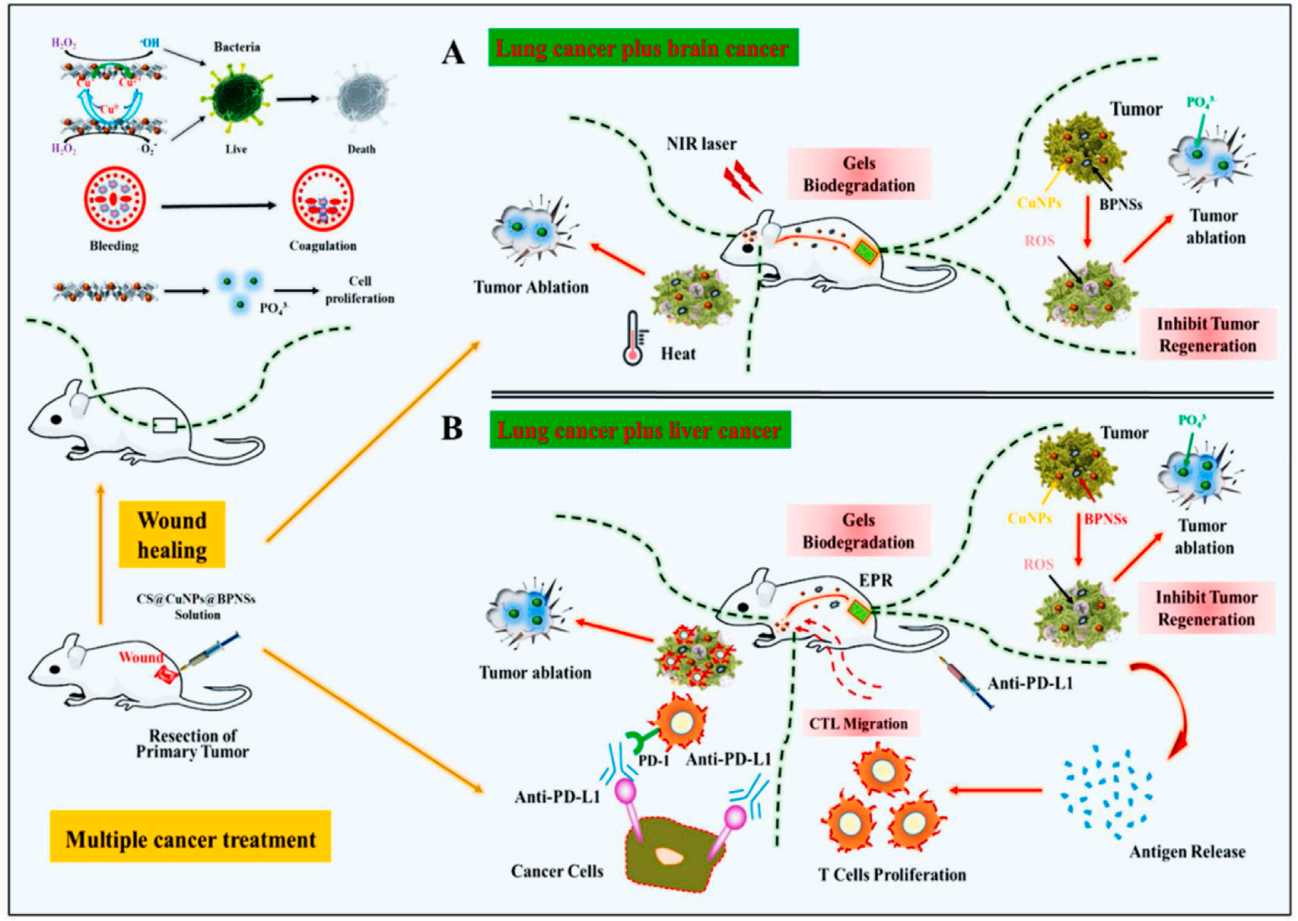
Illustration of the therapeutic mechanism of the hydrogel in inhibition of multiple cancer growth **(A)** Lung cancer with brain cancer **(B)** Lung cancer with liver cancer (Wang et al., 2021).

Wang et al. created a CS/CuNPs/black phosphate nanosheets (BPNSs) solution that is sustained-release, tumor microenvironment (TME) sensitive, and has superior photothermal and redox potential in the acidic TME (Figure 2). Under body temperature, this solution transforms into a biodegradable, spongy hydrogel. After surgery, the hydrogel condition within the body would significantly aid hemostasis and trap any remaining cancer cells in the blood. CuNPs would then take over the tumoricidal role by producing a sequence of redox reactions that would result in the formation of ROS, which could then kill any remaining cancer cells and stop the recurrence of an orthotopic tumor in order to simultaneously accomplish local antisepsis. The release of additional antigens, which significantly stimulates the systemic immune response, follows the cell death caused by the first chemo-dynamic treatment (CDT) action on the first primary tumor site throughout the therapeutic phase. The use of a PD-L1 enhances this response by protecting cytotoxic T lymphocyte (CTL) function. As a result, the activated CTLs and CDT impact of CuNPs might give synergistic treatment to the ectopic primary HCC tumor. The presence of BPNSs allowed biodegradable hydrogel fragments to pass the blood tumor barrier during NIR laser irradiation, resulting in the killing of brain cancer cells due to the CDT effect (Wang et al., 2021).

In order to create pH-degradable hydrogels, Gao et al. crosslinked carboxymethyl chitosan (CMCS) with an acid-labile ortho ester compound (OEDe). CMCS was subsequently cross-linked with a mixed cross-linker comprising OEDe and ethylene glycol diglycidyl ether (EGDE) at three different molar ratios in order to further adjust the pH-sensitivity and mechanical qualities. Then, hydrogels were wrapped around DOX-loaded gelatin nanoparticles with an average diameter of 50 nm to create a hybrid system that could be inserted into a tumor location in any shape for local chemotherapy. Intravenous injections of free DOX and DOX-loaded nanoparticles were tested for their ability to prevent tumor growth in mice harboring murine hepatoma tumors. The outcomes demonstrated that the cross-linked CMCS hydrogels could greatly extend the DOX release period as well as improve medication absorption into the tumor site. The hybrid hydrogels with the most pronounced anticancer activity were those with released DOX-loaded nanoparticles because they could further enhance the retention and permeation of DOX in tumor site. Therefore, the local therapy of solid tumors with pH-degradable hydrogels has significant potential (Gao et al., 2019).

Here in Table 1, we summarize clinical trials of chitosan.

**TABLE 1:** An overview for clinical trials of chitosan and derivatives.

ClinicalTrials.gov identifier	Conditions/Diseases	Status	References
NCT01278784	Dry Eye Syndromes	Phase 1, September 2011	https://clinicaltrials.gov/ct2/show/NCT01278784
NCT02323451	Osteoarthritis, Knee	Phase 4, April 2017	https://clinicaltrials.gov/ct2/show/NCT02323451
NCT02668055	Wounds	Phase 1, December 2015	https://clinicaltrials.gov/ct2/show/NCT02668055
NCT00454831	Hypercholesterolemia	Phase 2, September 2007	https://clinicaltrials.gov/ct2/show/NCT00454831
NCT0300765	Gynecologic Disease	Phase 3, August 2017	https://clinicaltrials.gov/ct2/show/NCT03007654
NCT01597817	Atopic Dermatitis	Phase 2, December,2012	https://clinicaltrials.gov/ct2/show/NCT01597817
NCT00521937	Diabetes Foot Ulcer	Phase 3, December,2010	https://clinicaltrials.gov/ct2/show/NCT00521937
NCT03202446	Breast Cancer Stage IIIA, IIIB and IV	Phase 3, January 2018	https://clinicaltrials.gov/ct2/show/NCT03202446
NCT03188289	Wisdom Teeth	Phase 4, September 2014	https://clinicaltrials.gov/ct2/show/NCT03188289
	Oral Surgery		

## 7 Discussion

Injectable hydrogels use their carrier capability and processing flexibility as an *in-situ* gelling regime. Although a sol-gel transition under moderate circumstances and well-controlled kinetics is required to provide a medical injection, the administration would also have minimal invasion and enable for filling irregularly shaped gaps. However, there are still a few fundamental issues to be concerned about to fully utilize injectable hydrogels in the biomedical area, aside from focusing on developing novel characteristics and sophisticated functionalities. First, while designating “new” materials for prospective clinical trials, it is important to consider the greater risk involved. The second topic is how a hydrogel’s properties could be impacted by the production process. Injectable gels must go through a sol-gel transition close to or at the desired insertion location, unlike *in situ* forming gels and 3D printing. As a result, the gelation shouldn't be too slow if the pregel isn't excessively viscous. Controlling the dosing of a hydrogel formulation could be challenging. Finding a solution with the right viscosity for injection, the necessary gelation kinetics, and the reliable mechanical characteristics of the harvested gel might not always be possible. The mechanical characteristics of the injected hydrogels are often poorer than those of the *in situ* generated ones, specifically for hydrogels with higher modulus or strength. Even though some clever approaches, such as the recently developed conjoined-network strategy, have been established for making high-performance hydrogels, a multistep synthesis procedure may reject the attempt to harvest such hydrogels by injection processing. However, given our focus on clinical users, we may anticipate more advancements in the creation of injectable hydrogels with improved and possibly “smart” features.
